# Auraptene, a Monoterpene Coumarin, Inhibits LTA-Induced Inflammatory Mediators via Modulating NF-*κ*B/MAPKs Signaling Pathways

**DOI:** 10.1155/2021/5319584

**Published:** 2021-11-16

**Authors:** Chih-Hsuan Hsia, Thanasekaran Jayakumar, Wan-Jung Lu, Joen-Rong Sheu, Chih-Wei Hsia, Periyakali Saravana Bhavan, Manjunath Manubolu, Wei-Chieh Huang, Yi Chang

**Affiliations:** ^1^Graduate Institute of Medical Sciences, College of Medicine, Taipei Medical University, Taipei 110, Taiwan; ^2^Translational Medicine Center, Shin Kong Wu Ho-Su Memorial Hospital, Taipei 111, Taiwan; ^3^Department of Pharmacology, School of Medicine, College of Medicine, Taipei Medical University, Taipei 110, Taiwan; ^4^Department of Medical Research, Taipei Medical University Hospital, Taipei 110, Taiwan; ^5^Graduate Institute of Metabolism and Obesity Sciences, College of Nutrition, Taipei Medical University, Taipei 110, Taiwan; ^6^Department of Zoology, Bharathiar University, Coimbatore 641046, Tamil Nadu, India; ^7^Department of Evolution, Ecology and Organismal Biology, Ohio State University, Columbus, OH 43212, USA; ^8^School of Medicine, Fu Jen Catholic University, New Taipei City 242, Taiwan; ^9^Department of Anesthesiology, Shin Kong Wu Ho-Su Memorial Hospital, Taipei 111, Taiwan

## Abstract

**Objective:**

Oxidative stress-mediated inflammatory events involve in the progress of several diseases such as asthma, cancers, and multiple sclerosis. Auraptene (AU), a natural prenyloxycoumarin, possesses numerous pharmacological activities. Here, the anti-inflammatory effects of AU were investigated in lipoteichoic acid- (LTA-) induced macrophage cells (RAW 264.7).

**Methods:**

The expression of cyclooxygenase (COX-2), tumor necrosis factor (TNF-*α*), interleukin-1*β* (IL-1*β*), and inducible nitric oxide synthase (iNOS) and the phosphorylation of extracellular signal-regulated kinase (ERK) 1/2, p38 MAPK, c-Jun N-terminal kinase (JNK), heme oxygenase (HO-1), p65, and I*κ*B*α* were all identified by western blotting assay. The level of nitric oxide (NO) was measured by spectrometer analysis. The nuclear translocation of p65 nuclear factor kappa B (NF-*κ*B) was assessed by the confocal microscopic staining method. Native polyacrylamide gel electrophoresis was performed to perceive the activity of antioxidant enzyme catalase (CAT).

**Results:**

AU expressively reduced NO production and COX-2, TNF-*α*, IL-1 *β*, and iNOS expression in LTA-stimulated cells. AU at higher concentration (10 *µ*M) inhibited ERK and JNK, but not p38 phosphorylation induced by LTA. Moreover, AU blocked I*κ*B and p65 phosphorylation, and p65 nuclear translocation. However, AU pretreatment was not effective on antioxidant HO-1 expression, CAT activity, and reduced glutathione (GSH, a nonenzymatic antioxidant), in LTA-induced RAW 264.7 cells.

**Conclusion:**

The findings of this study advocate that AU shows anti-inflammatory effects via reducing NF-*κ*B/MAPKs signaling pathways.

## 1. Introduction

Various chemicals and pathogens considered as harmful stimuli produce inflammation, which is a protective response of our body. Inflammation can be classified as acute and chronic, which induces pain and tissue injuries. Rapid onset and short duration of action can be noticed in the acute form, which is facilitated by the excretion of numerous cytokines including interleukin-1 (IL-1), IL-6, IL-11, IL-8, and tumor necrosis factor-alpha (TNF-*α*) [1, 2]. Nevertheless, in chronic inflammation, persistence of the inflammatory reactions could induce the migration of lymphocytes and macrophages to the damaged tissues [3]. Chronic inflammatory responses have been associated with the progression of various diseases such as asthma, arthritis, and neurodegenerative disorders [4]. Studies have established the involvement of several mediators including prostaglandin E2 (PGE2) in inflammatory events. Various symptoms including bone metabolism, wound healing, kidney function, blood vessel, and the immune responses have been associated with PGE2 secretion [5]. Cyclooxygenase (COX-2) protein can be expressed in response to physical, chemical, and biological stimulation [6]. The production of PGE2 can be augmented by COX-2, which denotes a central step in the events of inflammation.

Oxidative stress is known to be induced by elevated reactive oxygen species (ROS) and nitric oxide (NO) or reduced antioxidant enzymes catalase (CAT) and superoxide dismutase (SOD) and nonenzymatic glutathione (GSH) [7, 8]. Studies have indicated that oxidative stress plays a major role in the progress of inflammatory diseases [9]. The major component of Gram-positive bacteria, lipoteichoic acid (LTA), induces pathogenesis of sepsis [10] and lung injury by producing inflammatory reactions [11]. Therefore, examining the mechanisms that control LTA-stimulated cell activation is important for the analysis and treatment of lung inflammatory diseases. This bacterial component stimulates the release of IL-1*β*, IL-6, and TNF-*α* [12]. LTA induces TNF-*α* and IL-6 expressions by inducing the phosphorylation of ERK1/2 in macrophages, and it also activates nuclear translocation of nuclear factor- (NF-) *κ*B from the cytoplasm [13]. It has been proposed that various plant-based natural components have reported to have anti-inflammatory effects through suppressing inflammation-associated mediators and enhancing antioxidant defense molecules.

Auraptene, a geranyloxyl moiety of C-7 (7-geranyloxycoumarin), is a promising and most rich natural prenyloxycoumarin compound [14]. Plants of the Rutaceae family are the highest source of auraptene, and it is also the most general component of citrus fruits. Hence, citrus species are the major natural source of auraptene [14]. Several exciting pharmacological activities have been reported for this bioactive phytochemical such as antioxidant [15], anti-inflammatory [16], antimicrobial [17], antigenotoxic [18], neuroprotective [19], and immunomodulatory properties [20]. Murakami et al. [16] had well discussed the effect of auraptene in inflammation-mediated carcinogenesis. A study specified that dietary supplementation of auraptene in mice diminishes pulmonary metastasis of B16BL6 melanoma cells and prevents the growth of metastatic tumors in the lungs via inducing apoptosis [21]. In addition, auraptene showed promising effects of wound healing through inhibiting the secretion of inflammatory mediators in vitro, including IL-6 and IL-8 [22]. Hence, this study aimed to assess the anti-inflammatory mechanism of auraptene against LTA-stimulation in RAW 264.7 cells.

## 2. Materials and Methods

### 2.1. Materials

**Figure 1:**
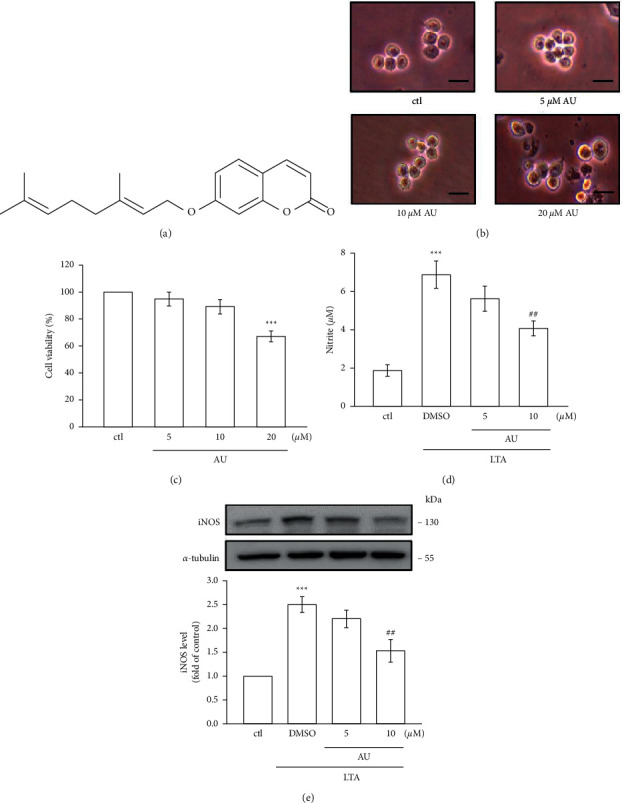
Chemical structure of auraptene (AU) and the effects of AU on morphology and cell viability and on LTA-induced NO production and iNOS expression in RAW 264.7 cells. (a) Chemical structure of AU. (b), (c) Cells were pretreated with AU (5, 10, or 20 *μ*M) for 24. Cell viabilities were determined by the MTT assay. Scale bar = 25 *μ*m. (d), (e) Cells were untreated or pretreated with AU (5 and 10 *μ*M) for 30 min prior to stimulation with LTA (5 *μ*g/ml) for 24 h. Control cells were not treated with LTA or AU. NO was measured using the Griess reaction assay. iNOS expression was detected using western blotting assay. The values shown are the means ± S.E.M. of four independent experiments. ^*∗∗∗*^*P* < 0.001 vs. the control cells; ^##^*P* < 0.01 vs. LTA-stimulated cells.

RAW 264.7 cells were obtained from the American Type Culture Collection (ATCC, Manassas, VA, USA, TIB-71). Auraptene (AU, >98%, Figure 1(a)) was purchased from ChemFaces Biochem, Wuhan, Hubei, China. Sigma (St Louis, MO, USA) supplied potassium ferricyanide, ferric chloride, and dimethyl sulfoxide (DMSO). Santa Cruz Biotechnology (Dallas, TX, USA) supplied anti-iNOS and COX-2 polyclonal antibodies (pAb). We purchased antibodies against TNF-*α*, phospho-p38 MAPK Thr180/Tyr182, phospho-c-JNK (Thr183/Tyr185), phospho-p44/p42 ERK (Thr202/Tyr204), phospho-I*κ*B*α* Ser32/36, and phospho-NF-*κ*B p65 (Ser536) pAbs from Cell Signaling (Beverly, MA, USA). Anti-IL-1*β* and anti-HO-1 pAbs were purchased from BioVision (Milpitas, CA, USA) and Enzo (Farmingdale, New York, USA), respectively. The antibody against *α*-tubulin was purchased from NeoMarkers (Fremont, CA, USA). AU was dissolved in 0.1% DMSO.

### 2.2. Cell Viability and Morphology of RAW Cells

RAW 264.7 cells were cultivated in Dulbecco's Modified Eagle's Medium (DMEM) at 37°C under 5% CO_2_ and 95% air. At a concentration of 1 × 10^5^ cells/well, they were pretreated with AU (5–20 *μ*M) for 24 h. The 3-(4,5-dimethylthiazol-2-yl)-2,5-diphenyl-2H-tetrazolium bromide (MTT) assay was used to measure cell viability in which 5 mg/mL of MTT working solution was added to the culture medium. The formation of crystals was digested by suing 300 *µ*l of DMSO. The formula of absorbance of treated cells/absorbance of control cells × 100% is used to measure the cell viability index.

### 2.3. Measurement of NO Production

To estimate the level of NO, AU at 5 and 10 *μ*M was added to cells with or without LTA (5 *μ*g/ml) for 24 h in the medium. Briefly, a 100 *µ*l equal volume of culture suspension and Griess reagent was mixed and incubated for 10 min. NO levels were estimated by quantifying nitrite levels by an MRX absorbance reader with the optical density at 550 nm.

### 2.4. Immunoblotting Assay

The equal amount (50 *µ*g) of proteins from 6 × 10^5^ cells were run on 12% sodium dodecyl sulphate-polyacrylamide gel electrophoresis (SDS-PAGE) gels. The separated proteins were transferred to polyvinylidene difluoride (PVDF) membranes and then blocked using 5% skim milk for 40 min. After blocking, the membrane was titrated with different primary antibodies of targeted proteins for 2 h and consequently incubated with anti-rabbit IgG or sheep anti-mouse IgG for 1 h. The intensity of protein bands was measured by using the Biolight Windows Application, V2000.01 (Bio-Profil, Vilber Lourmat, France) software.

### 2.5. Confocal Microscopy Assay

Cells were seeded at 5 × 10^4^/well, cultured on cover slips, and treated by AU (10 *μ*M) for 30 min and then triggered by LTA (5 *μ*g/ml) for 1 h. Coverslips were successively fixed with 4% paraformaldehyde for 10 min at 37°C, double washed using PBS, incubated with 0.1% Triton X-100 for 10 min, and then, blocked with 5% BSA for 1 h. Besides, the primary p65 antibody was added over the coverslips at 4°C overnight, and then, secondary goat anti-rabbit IgG antibody was incubated for 1 h at 37°C. 4,6-Diamidino-2 phenylindole (DAPI) was used to stain nuclei in cells. The location of nuclear translocation of p65 was spotted by using the Leica TCS SP5 confocal spectral microscope imaging system (Mannheim, Germany).

### 2.6. Detection of Antioxidant Enzyme Catalase (CAT)

According to the method defined by Woodbury et al. [23], a native polyacrylamide gel electrophoresis (NATIVE-PAGE) was run to spot the relative banding patterns of antioxidant enzyme catalase (CAT). To this analysis, unlike normal SDS-PAGE, the running buffers and protein samples did not heat and omit SDS. The equal amounts of 50 *μ*g proteins were run in 8% PAGE.

### 2.7. Statistical Analysis

The results are presented as mean ± standard error (S. E. M). The statistical difference among the groups was determined using one-way analysis of variance (ANOVA). Statistical alterations were detected significant. The *P* value of the Student–Newman–Keuls test was regarded as *P* < 0.05.

## 3. Results

### 3.1. AU Did Not Affect the Viability and Morphology of RAW 264.7 Cells

Cell morphology and viability were studied to evaluate the toxic effect of AU in RAW 264.7 cells. Among the tested concentrations of 5, 10, and 20 *μ*M AU in RAW cells for 24 h, 5 and 10 *μ*M did not affect cell morphology as well as viability (Figures 1(b) and 1(c)), respectively. However, AU at 20 *μ*M significantly affected the morphology and viability of RAW cells. Thus, AU at feasible concentrations of 5 and 10 *μ*M were used for the subsequent investigation.

### 3.2. LTA-Induced NO Production and iNOS Were Inhibited by AU

Griess reaction was applied to measure the level of NO production in AU pretreated LTA-induced RAW 264.7 cells. Systemic inflammatory events have been reported to induce a proinflammatory mediator NO [24]. A rate‐limiting enzyme, inducible nitric oxide synthase (iNOS), regulates the production of NO [25]. To examine if AU inhibits NO production via the modulation of iNOS expression, the expression of iNOS was detected as shown in Figure 1(e). Figures 1(d) and 1(e) show that, at a high concentration of 10 *μ*M, AU significantly inhibited the LTA‐induced production of NO and its enzyme iNOS expression (control: 1 ± 0, DMSO: 2.5 ± 0.2, 5 *μ*M: 2.2 ± 0.2, 10 *μ*M: 1.5 ± 0.2) in RAW 264.7 cells. This result apprehends that the inhibition of iNOS expression by AU may be involved in the inhibition of LTA‐induced NO production.

### 3.3. AU Inhibited LTA-Induced IL-1*β*, TNF-*α*, and COX-2 Expressions

**Figure 2:**
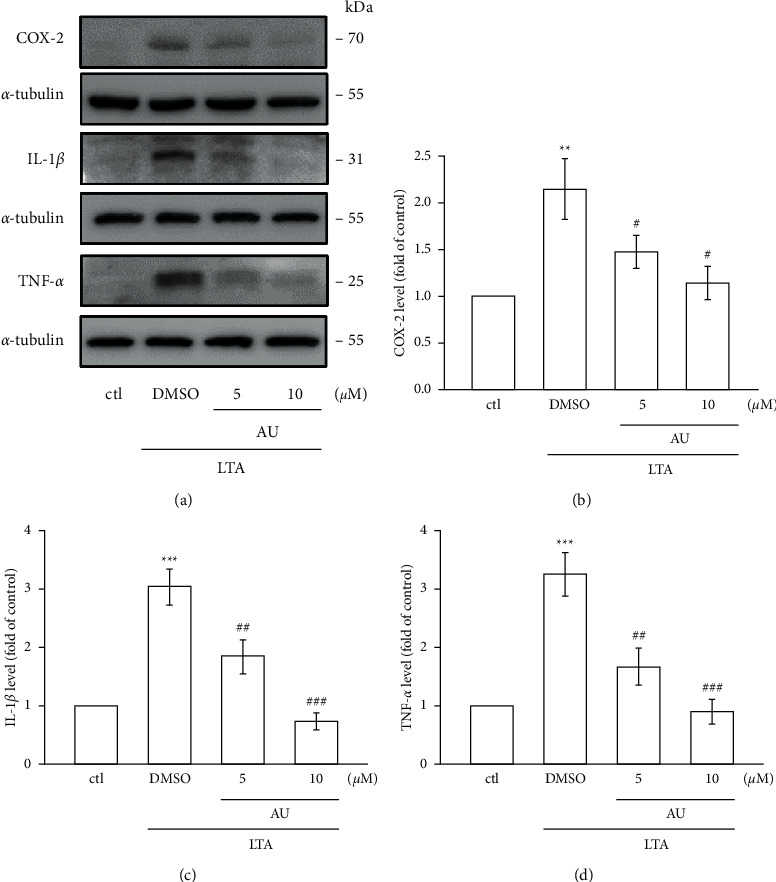
Effects of AU on the LTA-induced expression COX-2, IL-1*β*, and TNF*-α* in RAW 264.7 macrophages. (a)–(d) Cells were untreated or pretreated with AU (5 and 10 *μ*M) for 30 min and then stimulated with LTA (5 *μ*g/ml) for 24 h. COX-2, IL-1*β*, and TNF*-α* were detected as described in Section 2. The values shown are the means ± S.E.M. of four independent experiments. ^*∗∗*^*P* < 0.01 and ^*∗∗∗*^*P* < 0.001 vs. the control cells; ^#^*P* < 0.05, ^##^*P* < 0.01, and ^###^*P* < 0.001 vs. LTA-stimulated cells.

LTA stimulated the levels of COX-2 (2.1 ± 0.3, *P* < 0.01), IL-1*β* (3.1 ± 0.3, *P* < 0.001), and TNF-*α* (3.3 ± 0.4, *P* < 0.001) dramatically compared to the nonstimulated control RAW cells (Figures 2(a)–2(d)). In contrast, AU at 5 and 10 *μ*M distinctly alleviated COX-2 (5 *μ*M: 1.5 ± 0.2, 10 *μ*M: 1.1 ± 0.2), IL-1*β* (5 *μ*M: 1.9 ± 0.3, 10 *μ*M: 0.7 ± 0.1), and TNF-*α* (5 *μ*M: 1.7 ± 0.3, 10 *μ*M: 0.9 ± 0.2) induced by LTA. Moreover, AU more prominently inhibited IL-1*β* and TNF-*α* (Figures 2(c) and 2(d)).

### 3.4. AU Inhibits ERK1/2 and JNK1/2, But Not p38 MAPK Phosphorylation

**Figure 3:**
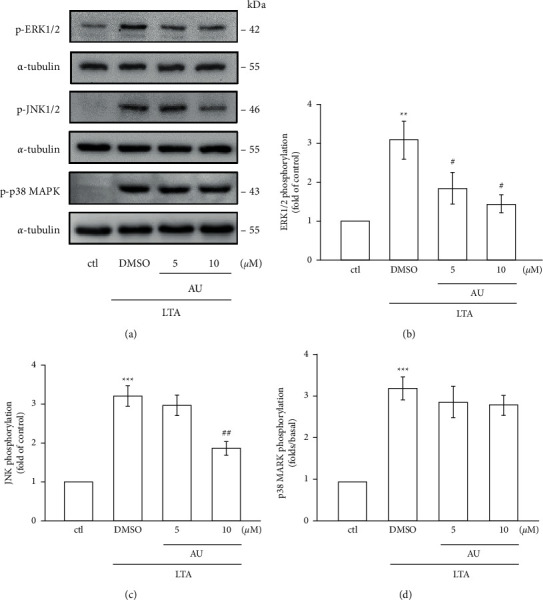
Effects of AU in LTA-induced phosphorylation of MAPKs in RAW 264.7 macrophages. (a) Cells were untreated or pretreated with AU (5 and 10 *μ*M) for 30 min and were then stimulated with LTA (5 *μ*g/ml) for 1 h. The specific pERK, pJNK, and p38 MAPK antibodies were used to detect these proteins. *α*-Tubulin was used as the internal control. (b)–(d) The statistical values shown are the means ± S.E.M. of four independent experiments. ^*∗∗*^*P* < 0.01 and ^*∗∗∗*^*P* < 0.001 vs. the control cells; ^#^*P* < 0.05 and ^##^*P* < 0.01 vs. LTA-stimulated cells.

We examined the effect of AU on LTA‐induced mitogen-activated protein kinases (MAPKs), since several studies have shown that these molecules actively involve on inflammation‐related events.  Figure 3 shows the elevated phosphorylation of ERK1/2 (3.1 ± 0.5), JNK1/2 (3.2 ± 0.3), and p38 MAPK (3.2 ± 0.3) in LTA-induced RAW cells compared to control cells. However, AU at a higher concentration of 10 *µ*M significantly diminished the LTA-induced phosphorylation of JNK1/2 (1.9 ± 0.2), and it concentration-dependently inhibited the ERK1/2 phosphorylation (5 *μ*M: 1.8 ± 0.4, 10 *μ*M: 1.4 ± 0.2); however, it is not effective on p38 (5 *μ*M: 2.9 ± 0.4, 10 *μ*M: 2.8 ± 0.2). These outcomes designated that AU reveals its inhibitory effects in LTA-induced inflammatory events in RAW 264.7 cells via suppressing ERK1/2 and JNK1/2 signaling cascade.

### 3.5. LTA‐Induced NF-*κ*B Signaling Pathway Was Inhibited by AU

**Figure 4:**
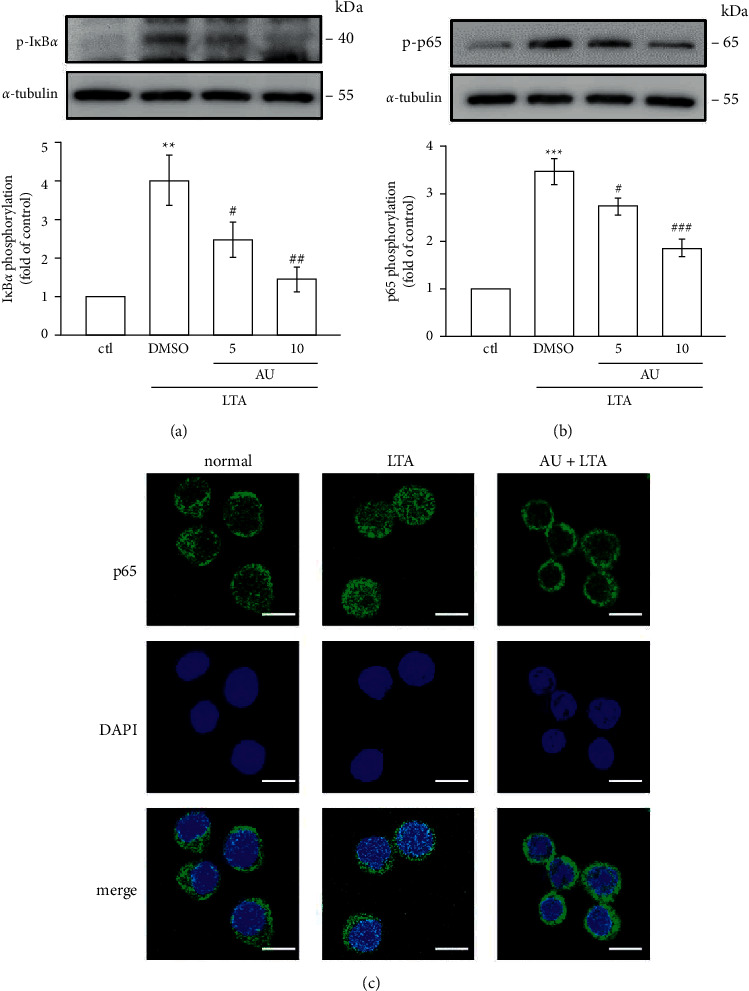
AU controls the NF-*κ*B signaling pathway induced by LTA in RAW 264.7 macrophages. Cells were pretreated with AU (5 and 10 *μ*M) for 30 min and were then stimulated with LTA (5 *μ*g/ml) for 1 h. The phosphorylation of (a) I*κ*B*α* and (b) p65 in LTA-induced RAW cells was detected as described in Section 2. (c) PTE inhibited LTA-induced p65 nuclear translocation. The values shown are the means ± S.E.M. of four independent experiments. ^*∗∗*^ < 0.01 and ^*∗∗∗*^ < 0.001 vs. the control cells; ^#^*P* < 0.05, ^##^*P* < 0.01, and ^###^*P* < 0.001 vs. LTA-stimulated cells.

NF‐*κ*B, a major transcription factor, is constantly inducing proinflammatory mediators and cytokines. This transcription factor translocates to the nucleus once it activates and binds with target DNA and then controls the activation of numerous inflammatory cytokines [25]. Here, the inhibitory effect of AU on NF‐*κ*B signaling pathways was examined by investigating the phosphorylations of I*κ*B*α* and p65 and also the nuclear translocation of p65 in LTA‐induced RAW cells. The results showed that AU reduced LTA‐induced I*κ*B*α* (DMSO: 4.0 ± 0.7, 5 *μ*M: 2.5 ± 0.5, and 10 *μ*M: 1.4 ± 0.3) and p65 phosphorylation (DMSO: 3.5 ± 0.3, 5 *μ*M: 2.7 ± 0.2, and 10 *μ*M: 1.9 ± 0.2) (Figures 4(a) and 4(b)) and withdrew the nuclear translocation of p65 (Figure 4(c)). These results demonstrate that AU's anti-inflammatory effect in LTA-induced cells may probably be via inhibiting the NF‐*κ*B signaling pathway.

### 3.6. AU Enhances Antioxidant Defense Molecules

**Figure 5:**
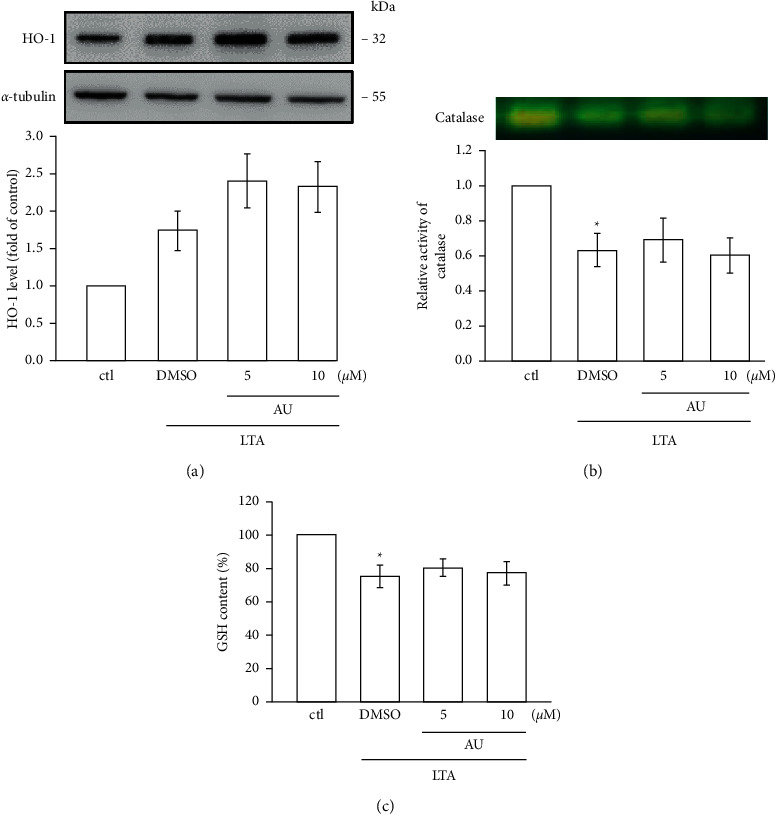
AU enhances antioxidant defense molecules in LTA-stimulated RAW cells. Cells were untreated or pretreated with AU (5 and 10 *μ*M) for 30 min followed by LTA (5 *μ*g/ml) for 24 h. The expression of HO-1 (a), catalase (CAT) activity (b), and glutathione (GSH) content (c) in LTA-induced RAW cells was determined using western blotting, native polyacrylamide gel electrophoresis (NATIVE-PAGE), and spectrophotometric analyses, respectively. The values shown are the means ± S.E.M. of four independent experiments. ^*∗*^*P* < 0.05 vs. the control cells.

Oxidative stress occurs by the elevated levels of reactive oxygen species (ROS) and NO or reduced levels of antioxidant defense molecules, such as reduced glutathione (GSH), catalase (CAT), and superoxide dismutase (SOD) [7]. Numerous studies have established that oxidative stress could induce the progress of inflammatory diseases [26]. LTA stimulation in RAW cells has been demonstrated to decrease in the expression of HO-1 (1.7 ± 0.3), antioxidant enzyme catalase, and the nonenzymatic GSH (Figures 5(a)–5(c)). AU pretreatment was not effective on LTA-stimulated reduction of HO-1 (5 *μ*M: 2.4 ± 0.4, 10 *μ*M: 2.3 ± 0.3), CAT, and GSH in RAW cells. These results indicate that the antioxidant defense systems could not play a role in AU-mediated anti-inflammatory effects in LTA-stimulated RAW cells.

## 4. Discussion

Auraptene (AU), a natural prenyloxycoumarin, is mostly present in citrus fruits. Auraptene (AU) possesses numerous pharmacological properties such as anticancer, antibacterial, antioxidant, and antiinflammatory [27]. Here, we found that auraptene (5 and 10 *μ*M) did not display cytotoxicity in both control and LTA-stimulated RAW cells. Hence, the ideal concentrations of 5 and 10 *μ*M of auraptene were used in this study. A study exposed that auraptene at concentrations of 5–40 *μ*M had no cytotoxicity on murine lymphocytes [28]. Together, as revealed in the present study, anti-inflammatory and antioxidative effects of auraptene are not through its cytotoxicity. Moreover, this study found that anti-inflammatory effects of AU was facilitated via preventing the production of NO and its enzyme iNOS expression. Auraptene also inhibited the LTA-induced protein expression of IL‐1*β* and TNF‐*α* by inhibiting the mitogen activated protein kinases (MAPKs)/NF‐*κ*B pathways.

As it is established, proinflammatory cytokines and mediators such as NO, IL-1*β*, IL-6, and TNF-*α* play a major role in the inflammatory process. Chronic inflammation has been reported to cause several diseases such as cancers, arthritis, and cardiovascular diseases [29]. A recent study specified that AU at 10–90 *μ*M reduced the levels of IL-6 and TNF-*α* in phytohemagglutinin- (PHA-) stimulated human lymphocytes [30]. A previous study from these authors has also established that AU alleviates IL-4, IL-10, and interferon (IFN-*γ*) levels [29]. NO plays a role in the pathogenesis of several inflammatory disorders, and its production in activated macrophages via the rate-limiting enzyme iNOS induces several acute and chronic inflammatory conditions [31]. COX-2 is reported to be overexpressed during the course of LPS-induced inflammatory reaction [32]. Studies have described that the overexpression of iNOS and COX-2 stimulates the activation of NO and PGE_2_ in activated macrophages, respectively. Overproduction of such inflammatory mediators can result in chronic inflammatory diseases [33]. Here, we found that AU expressively and without causing cytotoxicity inhibits the level of NO in LTA-stimulated RAW 264.7 cells. The AU's inhibitory effect on LTA-induced NO production appears to involve the reduction of iNOS expression. Moreover, AU dramatically inhibited the LTA-induced expression of iNOS, COX-2, TNF‐*α*, and IL‐1*β*. Okuyama et al. [34] showed that AU suppressed the LPS-induced expression of COX-2, IL-1*β*, and TNF-*α* in astrocytes isolated from the cerebral cortex of ICR mice. Niu et al. found an inhibitory mechanism for AU via IL-2, IFN-*γ,* and IL-4 in lymphocytes isolated from C57BL/6 mice [28]. These results are consistent with our results and evident of the anti-inflammatory properties of AU.

The induction of inflammatory mediators involves the activation of multiple signal transduction pathways, including mitogen-activated protein kinases (MAPKs) such as p38, ERK, and JNK [35]. It is reported that blocking p38, ERK, and JNK MAPK pathways could decrease iNOS and COX-2 expression and TNF-*α* and IL-1*β* production in macrophage inflammation [36]. The MAPK/NF-*κ*B signaling pathway was conveyed to play a vital role in the expression of TNF-*α*, IL-6, IL-1*β*, and COX-2 in many cell types [37]. Therefore, we examined the effect of AU on MAPK/NF-*κ*B pathway activation. Niu et al. found esculin significantly inhibited the activation of the MAPK pathway in LPS-induced peritoneal macrophages [38]. Guo et al. found both degradation and phosphorylation of I*κ*B*α* and activation of NF-*κ*B p65 stimulated by LPS are significantly controlled by imperatorin in RAW 264.7 macrophages [39]. Our recent study found that pterostilbene, a natural substance of blueberry and an analog of resveratrol, significantly inhibited the NF-*κ*B signaling pathway and ERK phosphorylation in RAW 264.7 cells [40]. Thus, it is proposed that coumarin derivatives may inhibit the MAPK/NF-*κ*B signaling pathway in LPS-induced inflammatory reaction. The results of this study consistently showed that AU strongly reversed the LTA-induced phosphorylation of JNK and ERK and the nuclear translocation of the p65 subunit. The induction of NF-*κ*B is controlled by I*κ*B kinase (IKK) complex activation, and IKK phosphorylates I*κ*B*α* and initiates ubiquitin-dependent I*κ*B*α* degradation [41]. This process could lead NF-*κ*B translocation to the nucleus, where it attaches to the promoter regions of the target gene and brings proinflammatory mediators such as iNOS, COX-2, TNF-*α*, and IL-6 [42]. The phosphorylation of I*κ*B and p65 can be induced by LTA, and it also can induce p65 translocation from the cytoplasm to nuclei [13]. LTA binds with toll-like receptor (TLR2), which in turn activates NF‐*κ*B and consequently translocated to nuclei from the cytoplasm [43]. Hence, these outcomes may propose that AU decreases LTA-induced inflammatory events in RAW cells via inhibiting the activation of JNK/ERK and NF‐*κ*B pathways.

Activated oxygen (O_2_^*∗*^) radicals are metabolized to H_2_O and successively converted to H_2_O_2_ by superoxide dismutase enzymes (SOD) and then to H_2_O by glutathione peroxidase or to H_2_O_2_ and O_2_ by catalases (CAT) [44]. A previous study found that irisin, a molecule secreted from skeletal muscle in response to physical exercise, plays a regulatory role in an immune system activity and can protect the cell from free-radical-induced cellular oxidative damage by the activation of antioxidative mechanisms [45]. Furthermore, a rise in HO-1 expression was identified to exert both antioxidant and anti-inflammatory effects [44]. HO-1 plays an important role in the protection of oxidative stress in chronic disease [46]. Furthermore, HO-1 has been reported to inhibit various inflammatory responses to exhibit its cellular protective role. Several antioxidants can induce HO-1 expression to cope oxidative damage, and thus, compounds that can activate HO-1 expression may be favorable in the treatment of oxidative damage. A natural anti-inflammatory compound curcumin was found to increase the activity of CAT to protect RAW cells from LPS-induced ROS damages [47]. Reduction of reduced glutation (GSH) had reported to lead the progress of several diseases, as GSH inhibits oxidative stress-induced cell damage [48]. Therefore, we examined whether AU can involve the downstream mechanism via interaction with HO-1 to its antioxidative action. However, AU did not augment HO-1, CAT, and GSH, which postulates that antioxidant mechanisms may not associate to AU's anti-inflammatory role in LTA-induced RAW cells.

## 5. Conclusions

This study shows the anti-inflammatory effects of auraptene via diminishing iNOS, COX-2, IL-1*β*, and TNF-*α* expression in LTA-induced RAW 264.7 macrophages. The inhibitory property of AU is mediating at least in part via inhibiting NF-*κ*B, along with the MAPK (JNK and ERK) pathway. Moreover, this study also found that AU's anti-inflammatory role was not depending on antioxidant mechanisms, as AU was not effective in HO-1, CAT, and GSH in the LTA-induced inflammatory RAW 264.7 cells.

## Data Availability

Data can be obtained from the corresponding author on reasonable request.
