# Corrigendum to “Auraptene, a Monoterpene Coumarin, Inhibits LTA-Induced Inflammatory Mediators via Modulating NF-*κ*B/MAPKs Signaling Pathways”

**DOI:** 10.1155/2022/9805697

**Published:** 2022-02-21

**Authors:** Chih-Hsuan Hsia, Thanasekaran Jayakumar, Wan-Jung Lu, Joen-Rong Sheu, Chih-Wei Hsia, Periyakali Saravana Bhavan, Manjunath Manubolu, Wei-Chieh Huang, Yi Chang

**Affiliations:** ^1^Graduate Institute of Medical Sciences, College of Medicine, Taipei Medical University, Taipei 110, Taiwan; ^2^Translational Medicine Center, Shin Kong Wu Ho-Su Memorial Hospital, Taipei 111, Taiwan; ^3^Department of Pharmacology, School of Medicine, College of Medicine, Taipei Medical University, Taipei 110, Taiwan; ^4^Department of Medical Research, Taipei Medical University Hospital, Taipei 110, Taiwan; ^5^Graduate Institute of Metabolism and Obesity Sciences, College of Nutrition, Taipei Medical University, Taipei 110, Taiwan; ^6^Department of Zoology, Bharathiar University, Coimbatore 641046, Tamil Nadu, India; ^7^Department of Evolution, Ecology and Organismal Biology, Ohio State University, Columbus, OH 43212, USA; ^8^School of Medicine, Fu Jen Catholic University, New Taipei City 242, Taiwan; ^9^Department of Anesthesiology, Shin Kong Wu Ho-Su Memorial Hospital, Taipei 111, Taiwan

In the article titled “Auraptene, a Monoterpene Coumarin, Inhibits LTA-Induced Inflammatory Mediators via Modulating NF-*κ*B/MAPKs Signaling Pathways” [1], the corresponding author's affiliations were listed incorrectly. The correct affiliation list is as shown above.
